# Distal appendicular skeletal involvement of diffuse large B-cell lymphoma on technetium-99m methylenediphosphonate bone scintigraphy and ^18^F-fluorodeoxyglucose positron emission tomography/computed tomography: a case report

**DOI:** 10.1186/s13256-017-1246-y

**Published:** 2017-04-04

**Authors:** Insook Park, Sungmin Kang

**Affiliations:** 1 Department of Oral and Maxillofacial Surgery, Catholic University of Daegu School of Medicine, 33 Duryugongwon-ro 17-gil, Nam-Gu, Daegu, 705-718 Korea; 2 Department of Nuclear Medicine, Catholic University of Daegu School of Medicine, 33 Duryugongwon-ro 17-gil, Nam-Gu, Daegu, 705-718 Korea

**Keywords:** Appendicular bone involvement, Technetium-99m methylenediphosphonate bone scintigraphy, ^18^F-fluorodeoxyglucose positron emission tomography/computed tomography, Diffuse large B-cell lymphoma

## Abstract

**Background:**

We report a case of a patient with appendicular bone involvement of diffuse large B-cell lymphoma visualized by whole-body technetium-99m methylenediphosphonate bone scintigraphy (bone scan) and ^18^F-fluorodeoxyglucose positron emission tomography/computed tomography.

**Case presentation:**

A 73-year-old Asian man who had gingival swelling of the labial area of the left maxillary lateral incisor presented to our institution. Positron emission tomography/computed tomography demonstrated hypermetabolic lesions with maximum standardized uptake values of 12.15 in the left testis, 1.92 in the skin of the right chest, and 2.88 in the left ulna and third metatarsal bone. A bone scan showed multiple uptakes in the left ulna, hand, both tibiae, and the left foot. We diagnosed the tumor as diffuse large B-cell lymphoma by excision. Magnetic resonance imaging showed enhanced signaling of lesions with soft tissue edema in the olecranon of the left ulna, the third metacarpal bone of the left hand, and the third metatarsal bone of the left foot. Magnetic resonance imaging findings prompted a diagnosis of lymphoma. Eight cycles of chemotherapy plus external radiotherapy targeted to the involved bone sites were given for 5 months. Follow-up positron emission tomography/computed tomography and bone scanning revealed the disappearance of hypermetabolism and decreased uptake in lesions compared with the previous images. Laboratory data were also all within the normal limits after chemotherapy.

**Conclusions:**

This report highlights that appendicular bone involvement of diffuse large B-cell lymphoma can be detected on whole-body bone scans and by positron emission tomography/computed tomography.

## Background

Malignant lymphoma of bone accounts for 7% of all bone malignancies and 5% of extranodal lymphomas [1]. Reported bone involvement of diffuse large B-cell lymphoma (DLBL) is in the femur (27%), pelvis (15%), tibia/fibula (13%), polyostotic (13%), humerus (12%), spine (9%), other (5%), mandible (2%), radius/ulna (1%), scapula (1%), and skull (1%). Uncommonly, bones of the hands and feet are involved [2].

Technetium-99m methylenediphosphonate bone scintigraphy (bone scan) is a cost-effective and useful tool in a variety of diseases, and it is the most commonly used means of detecting bone metastasis, with variable diagnostic sensitivity and comparatively low specificity [3]. ^18^F-fluorodeoxyglucose ([^18^F]-FDG) positron emission tomography/computed tomography (PET/CT) is a standard diagnostic modality in the workup of many malignancies. Because malignant cells exhibit increased proliferation and glucose metabolism, PET/CT scans noninvasively detect a primary lesion and cancer metastasis in images by increased [^18^F]-FDG uptake [4]. This case report describes the detection of appendicular bone involvement of DLBL on whole-body bone scans and PET/CT scans.

## Case presentation

**Fig. 1:**
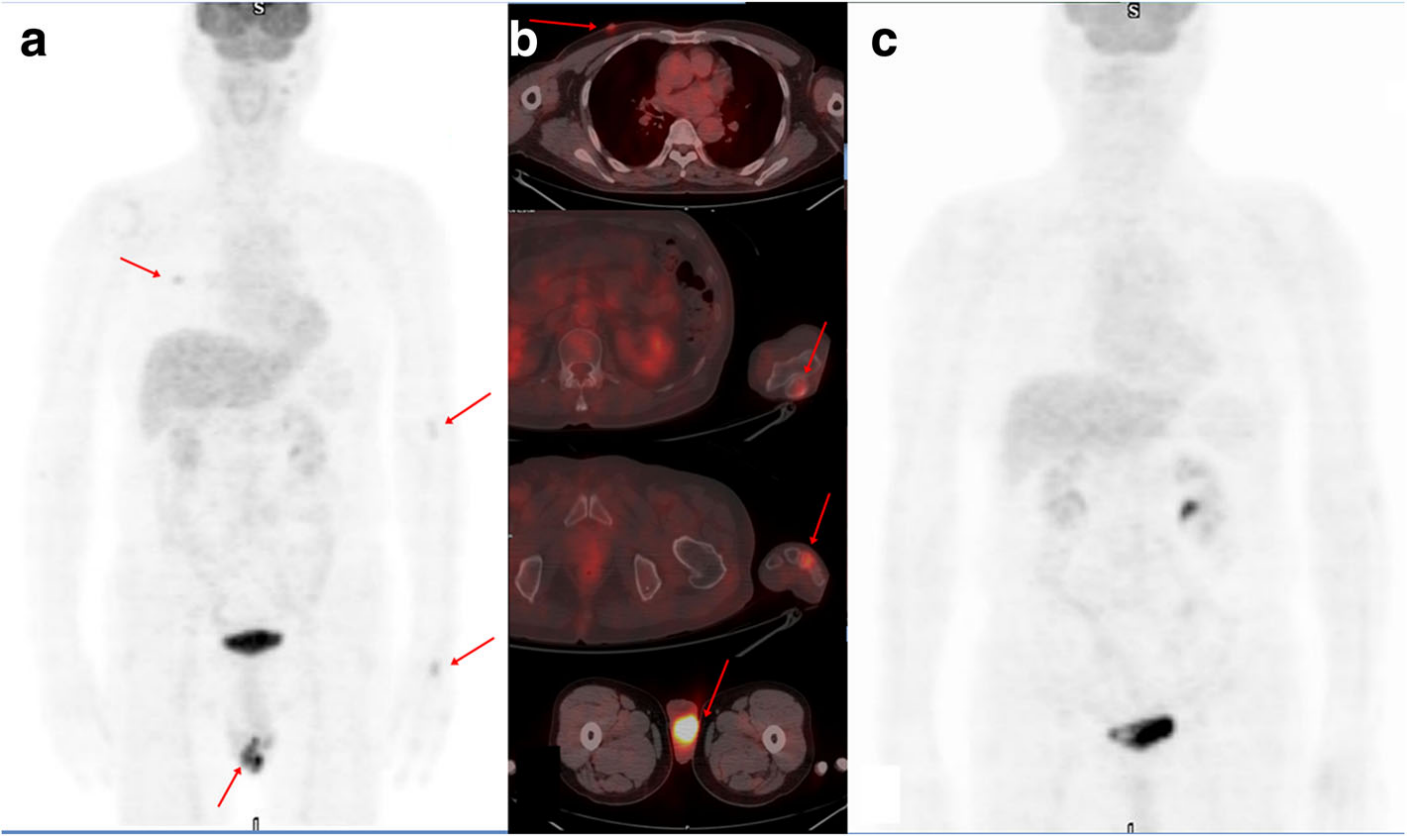
Images of a 73-year-old Asian man who underwent ^18^F-fluorodeoxyglucose positron emission tomography/computed tomography for metastasis evaluation for malignant lymphoma. **a** and **b** Maximum-intensity projection images obtained by ^18^F-fluorodeoxyglucose positron emission tomography/computed tomography shows focal hypermetabolic lesions (maximum standardized uptake values 1.92 [chest wall], 2.56 [olecranon of left ulna], 2.88 [left third metacarpal bone], and 12.15 [left testis]; *red arrows*), which were proven to be malignant lymphoma by histological and radiological examinations. **c** Follow-up ^18^F-fluorodeoxyglucose positron emission tomography/computed tomography after chemotherapy shows disappeared hypermetabolic lesions in the above-described sites

**Fig. 2:**
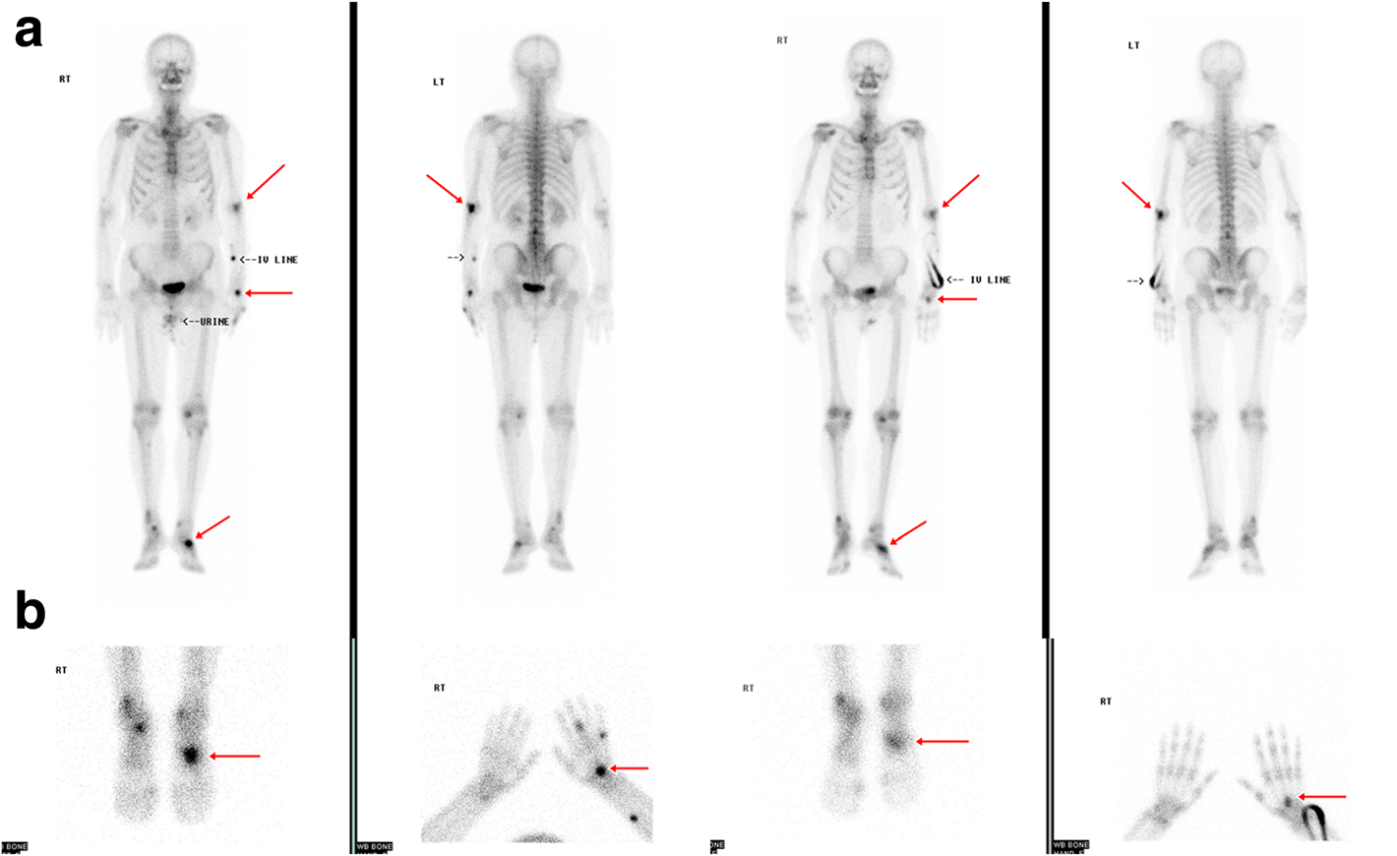
**a** Technetium-99m bone scintigraphy shows increased uptake in the olecranon of the left ulna, as well as the left hand and foot (*red arrows*). **b** Follow-up technetium-99m bone scintigraphy after chemoradiotherapy shows decreased uptake in the third metacarpal bone of the left hand (*red arrow*)

**Fig. 3:**
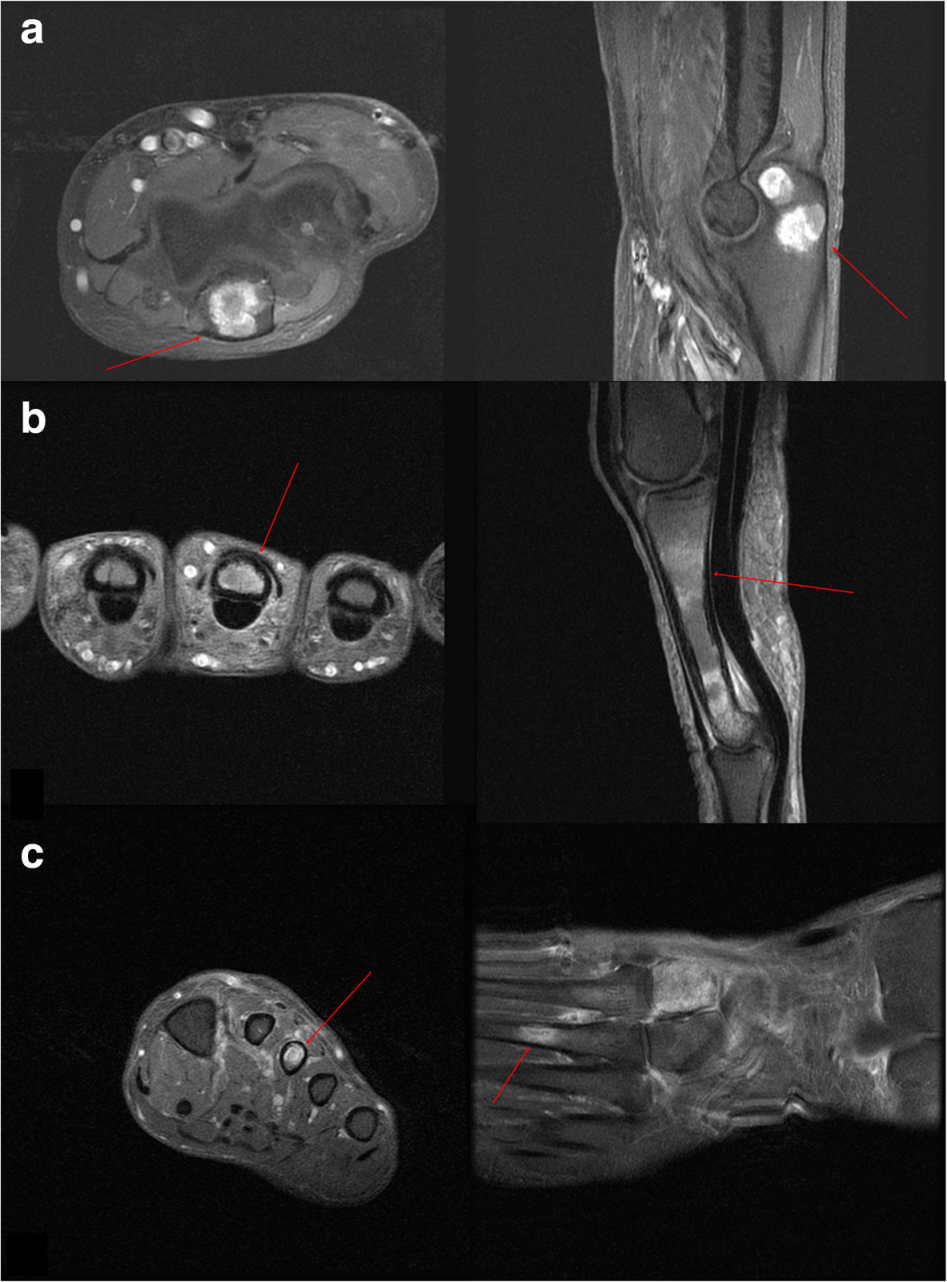
**a** Axial and sagittal T2-weighted magnetic resonance images show an increased signal mass in the olecranon of the left ulna (*red arrow*). **b** Axial and sagittal T2-weighted magnetic resonance images show an increased signal mass in the third metacarpal bone of the left hand (*red arrow*). **c** Axial and coronal T2-weighted magnetic resonance images show an increased signal mass in the third metatarsal bone of the left foot (*red arrow*)

A 73-year-old Asian man who had gingival swelling of the labial area of the left maxillary lateral incisor presented to our institution. Initially, it was suspected of being a radicular cyst in the left maxillary sinus incisor. No other symptoms, including fever, cough, or dyspnea, were present. The patient had no family history of cancer. Laboratory data, including tumor markers and peripheral blood appearance, were all within the normal limits. The patient underwent excision of the swelling. A pathological examination revealed a highly pleomorphic large-cell proliferation. Immunohistochemistry was diffusely positive for cluster of differentiation (CD) 20 and B-cell lymphoma 6 proteins. The patient’s Ki-67 index was approximately 80%. His tumor cells were negative for CD3, CD10, and creatine kinase. We diagnosed the tumor as DLBL with an immunohistological staining pattern consistent with germinal center B-cell derivation. PET/CT was performed to determine the stage. Increased FDG uptake in multiple masses and nodular lesions was evident, with maximum standardized uptake values of 12.15 in the left testis, 1.92 in the skin of the right chest, and 2.88 in the left ulna and the third metatarsal bone (Fig. 1a and b). A bone scan performed to diagnose the bone lesions revealed multiple uptakes in the left ulna, hand, both tibiae, and the left foot (Fig. 2a). On the basis of the PET/CT result, left orchiectomy was performed for accurate histological diagnosis. The lesion was confirmed as DLBL. Axial and sagittal T2-weighted magnetic resonance imaging (MRI) showed enhanced signaling of lesions with soft tissue edema in the olecranon of the left ulna (Fig. 3a), the third metacarpal bone of the left hand (Fig. 3b), and the third metatarsal bone of the left foot (Fig. 3c). MRI findings prompted a diagnosis of lymphoma. Eight cycles chemotherapy were given according to the rituximab, cyclophosphamide, doxorubicin, vincristine, and prednisone protocol plus external radiotherapy targeted to the involved bone sites for 5 months. Follow-up PET/CT (Fig. 1c) and bone scan (Fig. 2b) revealed the disappearance of hypermetabolism and decreased uptake in lesions compared with the previous images. Laboratory data were also all within the normal limits after chemotherapy.

## Discussion

DLBL comprises a group of large, lymphoid B-cell malignant proliferations that is clinically, morphologically, and genetically heterogeneous. It constitutes about 30% of all non-Hodgkin lymphomas and is the most common histologic subtype [5]. It is also characterized by relatively frequent extranodal presentation. The most common extranodal localizations are the stomach, central nerve system, bone, testis, and liver [6]. Presently, only extranodal areas affecting gingival areas of the left maxillary lateral incisor, the chest wall, the testis, and distal appendicular bone were affected with DLBL.

Bone scan is widely available and commonly used to detect bone metastases from cancers because it is inexpensive and sensitive and is able to capture a whole-body image [7]. Notably, PET/CT also provides whole-body images with an overall assessment of disease extent, and so can reveal lesions that are hidden on other conventional images. PET/CT has several advantages in the management of patients with lymphoma during staging and follow-up [8]. In our patient, DLBL was first diagnosed in the gingival area of the left maxillary lateral incisor, but unrevealed lymphoma involvement on the chest wall, testis, and distal appendicular bone were subsequently detected by whole-body bone scanning and PET/CT. PET/CT also is important in the early prediction of response to chemotherapy and the evaluation of residual masses after chemotherapy or radiation therapy [9, 10]. In our patient, decreased uptake visualized by bone scanning and PET/CT was apparent after chemo- and radiotherapy.

Malignant lymphoma of bone is classified into four subtypes: primary bone lymphoma (PBL), multifocal PBL, and disseminated lymphoma with secondary osseous involvement either within 6 months of lymphoma diagnosis via soft tissue/nodal disease or more than 6 months after diagnosis [11]. PBL is defined as lymphoma that is confined to the bone or bone marrow without evidence of concurrent systemic involvement, and it has the best prognosis of all primary bone malignant lesions [12]. PBL is commonly found in the appendicular skeleton, typically affecting the metadiaphysis of the femur, tibia, and humerus, and it has a better prognosis than disseminated disease with secondary osseous involvement [11]. Secondary osseous lymphoma is usually due to DLBL [11, 13] and preferentially affects the axial skeleton, such as the spine, pelvis, skull, ribs, and facial bones [13]. Secondary osseous lymphoma of distal appendicular bone is extremely rare. In our patient, DLBL was detected in the gingival area, but multiple lymphomatous involvement on the chest wall, testis, and distal appendicular bone was detected by bone scanning and PET/CT. Despite the improvement of disease by chemo- and radiotherapy, a poor prognosis is expected.

## Conclusions

Secondary DLBL involvement of distal appendicular bone is extremely rare. Bone scanning and PET/CT are important in the diagnosis and response assessment of DLBL involvement of appendicular bone, as in our patient. Bone scanning and PET/CT may be instrumental in establishing a diagnosis of lymphomatous involvement of distal appendicular bone.

## Abbreviations

Bone scan: Technetium-99m methylenediphosphonate bone scintigraphy
CD: Cluster of differentiation
DLBL: Diffuse large B-cell lymphoma
[^18^F]-FDG: ^18^F-fluorodeoxyglucose
MRI: Magnetic resonance imaging
PBL: Primary bone lymphoma
PET/CT: Positron emission tomography/computed tomography
